# What predicts collective dedication in health professions education? A path analysis among health and social care students

**DOI:** 10.1007/s10459-025-10485-w

**Published:** 2025-11-03

**Authors:** John Ian Wilzon T. Dizon, Qing He, Xiaoai Shen, Ma. Jenina Nalipay, Runjia Wang, Karen Man Kei Chan, Linda Chan, Jody Kwok Pui Chu, Amy Yin Man Chow, Julienne Jen, May P. S. Lam, Feona Chung Yin Leung, Mary Lok Man See, Dana Vackova, Pauline Pui Ning Yeung, George L. Tipoe, Fraide A. Ganotice

**Affiliations:** 1 Bau Institute of Medical and Health Sciences Education, Li Ka Shing Faculty of Medicine, The University of Hong Kong, Hong Kong, SAR China; 2 Department of Curriculum and Instruction, The Chinese University of Hong Kong, Hong Kong, SAR China; 3 Swallowing Research Laboratory at the Faculty of Education, The University of Hong, Hong Kong, China; 4 Department of Family Medicine and Primary Care and at the Bau Institute of Medical and Health Sciences Education, Li Ka Shing Faculty of Medicine, The University of Hong Kong, Hong Kong, China; 5 Department of Pharmacology and Pharmacy, Li Ka Shing Faculty of Medicine, The University of Hong Kong, Hong Kong, China; 6 Department of Social Work and Social Administration, Faculty of Social Sciences, The University of Hong Kong, Hong Kong, China; 7 Department of Professional Legal Education, Faculty of Law, The University of Hong Kong, Hong Kong, China; 8 School of Chinese Medicine, Li Ka Shing, Faculty of Medicine, The University of Hong Kong, Hong Kong, China; 9 School of Nursing, Li Ka Shing Faculty of Medicine, The University of Hong Kong, Hong Kong, China; 10 School of Public Health, Li Ka Shing Faculty of Medicine, The University of Hong Kong, Hong Kong, China; 11 School of Clinical Medicine, Li Ka Shing Faculty of Medicine, The University of Hong Kong, Hong Kong, China

**Keywords:** Interprofessional education, Collective dedication, Collective efficacy, Health professions students, Path analysis

## Abstract

**Background:**

In health professions education, cultivating commitment to collaborative practice is essential. However, collective dedication as a desired outcome in interprofessional education (IPE) often remains overlooked. Psychological factors contributing to team members’ collective dedication are poorly understood within health professions collaborative learning environments. This study examined relationships among team psychological factors (interdependence, relatedness, efficacy, and potency) and their influence on collective dedication in an IPE context.

**Method:**

Data were from 236 undergraduate students (Chinese medicine, Law, Medicine, Nursing, Pharmacy, Speech Therapy, and Social Work) who attended an IPE simulation at a higher education institution in Hong Kong. Participants completed a set of standardized questionnaires adapted to the IPE context, assessing positive interdependence, sense of relatedness, collective efficacy, group potency, and collective dedication. The data were analyzed using correlational and path analysis.

**Results:**

Findings showed that positive interdependence positively predicted students’ sense of relatedness, collective efficacy, and group potency. Further, sense of relatedness positively predicted collective efficacy, group potency, and collective dedication. Further, collective efficacy and group potency positively predicted collective dedication. Lastly, collective efficacy mediated the association between group potency and collective dedication.

**Conclusions:**

This study advances health professions education by examining the pathways to collective dedication in IPE. Positive interdependence indirectly affects collective dedication through students’ sense of relatedness, group potency, and collective efficacy. The findings provide practical implications of the findings for health professions educators and IPE program implementers.

**Supplementary Information:**

The online version contains supplementary material available at https://doi.org/10.1007/s10459-025-10485-w.

## Introduction

In higher education, academic institutions are earnestly persuaded to equip students with 21st-century collaboration-focused competencies (i.e., competencies that involve working together to jointly plan, implement, and solve problems through shared information, resources, and responsibilities (Camarihna-Matos & Afsarmanesh, 2008; Everett, 2008; James Jacob, 2015; Levin & Greenwood, 2008; Reed et al., 2021; Stentoft, 2017). This is in recognition of the desired complementarity of professional and interprofessional competencies needed to thrive in 21st-century workplaces. In the context of healthcare practice, the contemporary approach is promoted, whereby multiple health and social care professions work in synergy to manage patients’ complex healthcare needs. Hence, health professionals are not only expected to be experts within their fields of professional practice but are also required to demonstrate their interprofessional expertise by collaborating with others to work as a team in health care management. Interprofessional education (IPE), defined as an occasion when people from two or more professions learn *from*, *with*, and *about* each other (Barr, 2002; Center for Advancement of Interprofessional Education [CAIPE], 2002), has become a means to break down silos in education within which health and social care students are trained to be better interprofessional collaborators (Whitehead, 2007). Recent systematic reviews continue to demonstrate the positive impact of IPE on collaborative practice readiness and patient outcomes (Lutfiyya et al., 2019; Reeves et al., 2016; Saragih et al., 2023). To guide IPE development, the Interprofessional Education Collaborative (IPEC) has identified four core competency domains: values and ethics, roles and responsibilities, communication, and teams and teamwork (Interprofessional Education Collaborative, 2023).

Within the teams and teamwork domain, scholars have identified the need to disentangle the mechanisms of successful collaboration (Berger-Estilita et al., 2020; Bogossian et al., 2023; Oudbier et al., 2024). In particular, extant literature suggests that true IPE necessitates sustained members’ collective dedication, defined as the demonstration of a “sense of significance, enthusiasm, inspiration, pride, challenge, and absorption and refers to being fully concentrated and engrossed in one’s work” (Salanova et al., 2003, p. 48), to work effectively in a collaborative team context (Brazeau, 2013). Collective dedication among team members is essential for teams to attain successful outcomes, as teams tend to achieve the most when members share the same goals and demonstrate collective dedication.

Despite the growing literature on collective dedication in industries and organizations, collective dedication has received insufficient attention, and its attainment remains poorly understood in the IPE context. Hence, the IPE community of practice must identify factors that account for team collective dedication and the underlying psychological mechanisms or pathways that explain its achievement. Further, the IPE community remains unaware of actionable knowledge that can be used to craft effective policies and practices for optimizing collaboration outcomes. To address these limitations, the present study aims to investigate the relationships among team psychological factors (interdependence, relatedness, efficacy, and potency) and how they lead to collective dedication in a collaborative learning context. Specifically, we draw on the social interdependence theory (SIT; Deutsch, 1949) and the input-mediator-output (IMO) framework (McGrath, 1964) as guides, both of which will be discussed in detail in the succeeding sections, to examine a model of collective dedication. We propose that team members’ positive interdependence (input) would predict relatedness (motivation state), which in turn would predict team potency and collective efficacy (team emergent states), and subsequently, collective dedication (learning outcome) in the context of IPE. A summary of the study variables’ definitions can be found in Table 1.

**Table 1:** Summary of the definitions of the study variables

Variable name	Definition	Reference
Collective dedication	A sense of significance, enthusiasm, inspiration, pride, challenge, and absorption and refers to being fully concentrated and engrossed in one’s work in a collaborative team context.^*^	Salanova et al. (2003)
Collective efficacy	A group’s shared beliefs regarding their joint capabilities to plan and perform actions that lead to the achievement of their goals.	Bandura (1997)
Positive interdependence	Occurs when individuals perceive that the achievement of their goals is mutually dependent on each other’s contribution.	Deutsch (1949); Johnson and Johnson (2009)
Sense of relatedness	Refers to the feeling of being connected and important among others and to social organizations beyond oneself.	Furrer and Skinner (2003); Ryan & Deci (2000)
Group potency	The team’s generalized beliefs regarding their capabilities across various tasks and contexts (e.g., the belief that the team will be successful regardless of the task).	Gully et al. (2002)

Note: * = This construct is measured at the team level as shared dedication

### Potential factors influencing collective dedication

One of the well-founded theories that explains student interaction in collaborative learning is the social interdependence theory (Gully et al., 2002; Johnson & Johnson, 1999, 2009; Slavin, 1996) SIT states that team members will be motivated to cooperate when they perceive positive interdependence (Deutsch, 1949, 1973; Johnson, 2003), construed to exist when individuals perceive that the achievement of their goals is mutually dependent on each other’s contributions. Previous studies show that positive interdependence can enhance team effectiveness (Courtright et al., 2015; Widianto et al., 2024). Further evidence suggests that collaborative learning among students fosters more positive attitudes toward learning than competitive or individualistic learning (Johnson & Johnson, 1999). Owing to the shift in emphasis from a single profession to an interprofessional approach to care management, healthcare is one of the sectors where positive interdependence has been rapidly required (Frenk et al., 2010). Health professions, unlike other sectors, have to build more complex relationships, such as those between intraprofessional and interprofessional care providers, trainees and trainers, and patients. In the context of IPE, healthcare students work and collaborate to achieve a given goal. This collaboration enhances their ability to provide quality healthcare services. Despite this recognition, the mechanism that links positive interdependence and collective dedication remains poorly understood.

**Fig. 1:**
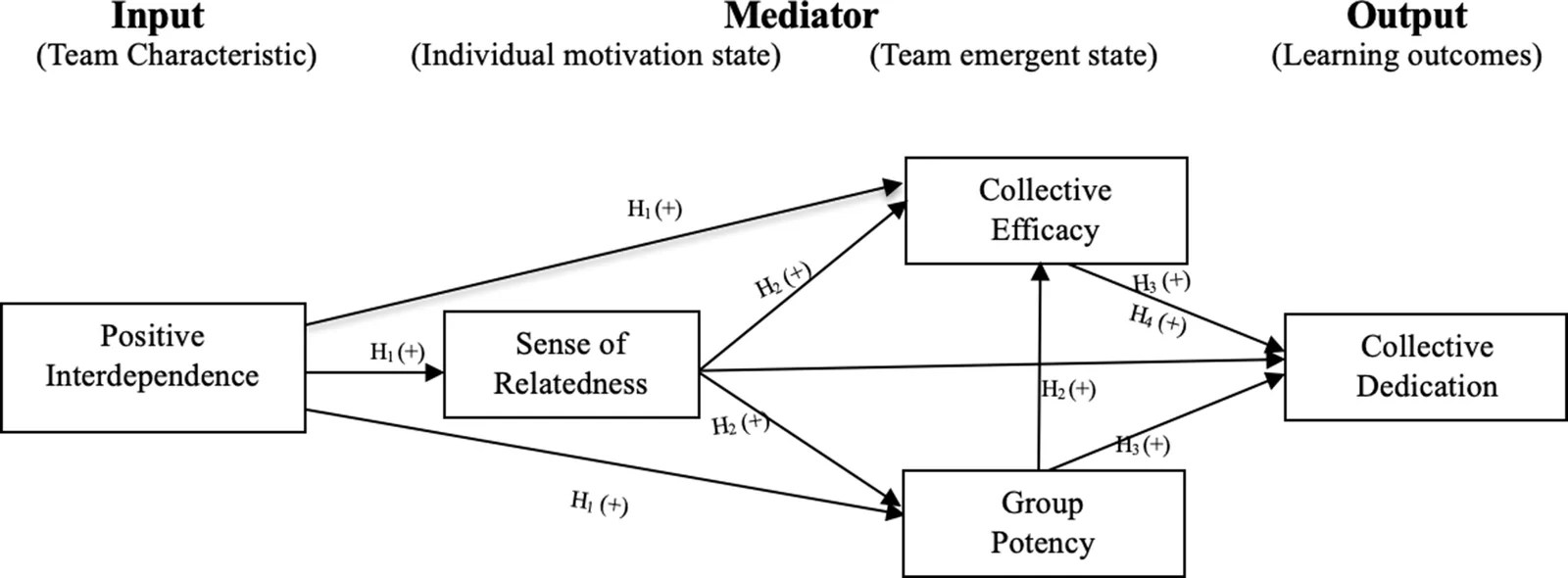
The hypothesized model of the relationship between positive interdependence, sense of relatedness, group potency, collective efficacy, and collective dedication. H = hypothesis, + = positive relations

Aside from the SIT, the input–mediator–output (IMO) framework (McGrath, 1964) is another popular model that is used for studying team processes. Specific to this model is that inputs are highlighted as antecedent factors that enable and constrain members’ interactions (Mathieu et al., 2008,), which are the team characteristics. In the same model, mediators are interpreted as team processes and emergent states that link inputs to output, while outputs are considered as results and by-products of team activity that are valued by one or more constituencies (Mathieu et al., 2008), which may include performance and affective reactions. Using the theoretical underpinnings of SIT and IMO, we conceptualized an indirect effect model (Fig. 1), elucidating the potential mechanism for the achievement of collective dedication in IPE.

### Team characteristics: positive interdependence

Given that collaborative learning is central to the IPE context, positive interdependence is essential for productive cooperative learning (Shimizu et al., 2020) to influence collective dedication. For this reason, fostering members’ interdependent attitudes is a team-desired attribute. Therefore, understanding why and how interdependence influences team collaboration outcomes in IPE is important, as it prepares students to form interdependent relationships by learning from and supporting other professions, as well as teaching and supporting them (Barr, 2002). Individual members become aware of their responsibilities when positive interdependence is developed (Shimizu et al., 2020). Given its interpersonal nature, it may also foster a higher sense of relatedness, a universal need to connect with other human beings, (Ryan & Deci, 2000) among the team members, as well as a higher level of collective efficacy and group potency, which makes individuals more inspired to demonstrate collective dedication in teamwork.

### Motivation state: the role of sense of relatedness

The sense of relatedness refers to the feeling of being connected and important among others and to social organizations beyond oneself (Furrer & Skinner, 2003; Ryan & Deci, 2000). Research in the educational setting has shown that a sense of relatedness can enhance school performance and other positive educational outcomes (Furrer & Skinner, 2003; Niemiec & Ryan, 2009). According to the SIT, positive interdependence is expected to drive team processes, particularly individual motivation states such as sense of relatedness. The degree of positive interdependence (e.g., common goals, functional aspect of team functioning) influences the perception that a group is a unified and coherent whole in which the members are bonded together (Johnson & Johnson, 2005). The perception of interdependence of a unified and coherent group may then foster a sense of relatedness (an emotional aspect of team functioning). Indeed, self-determination theory has identified the satisfaction of the innate psychological need for relatedness, along with autonomy and competence, as an important antecedent for individuals to achieve intrinsic motivation to work (Deci & Ryan, 2000). This motivation may, in turn, drive desirable cooperative learning outcomes, such as a collective dedication to collaborative learning (Ganotice et al., 2021).

### Team emergent states: the role of group potency and collective efficacy

Emergent states refer to the motivational, cognitive, and affective states of a team. These states may vary depending on the team context, inputs, processes, and output. Emergent states can be considered as both team inputs and proximal outputs (Marks et al., 2001). Research suggests that emergent states such as group potency and collective efficacy increase team effectiveness and performance (Kozlowski & Ilgen, 2006; Rapp et al., 2021; Stajkovic et al., 2009). Group potency is defined as the team’s generalized beliefs regarding their capabilities across various tasks and contexts (e.g., the belief that the team will be successful regardless of the task; Gully et al., 2002). A meta-analysis (Stajkovic et al., 2009) identified that group potency (a general belief with enduring temporal focus and broad outcome emphasis) likely operates through collective efficacy (a proximal belief with specific temporal focus and sensitivity to specific situations). On the other hand, collective efficacy is identified as a group’s shared beliefs regarding their joint capabilities to plan and perform actions that lead to the achievement of their goals (Bandura, 1997). Collective efficacy belief is consistent with social cognitive theory, which states that a higher sense of collective efficacy could lead to better team performance (Bandura, 1997). Collective efficacy is a strong predictor of team performance at both individual and group levels (Lent et al., 2006).

The sense of relatedness is expected to account for group potency and collective efficacy, which take place if one is emotionally attached to teammates. We can assume that the stronger the potency and collective efficacy among teammates, the more willing they are to dedicate themselves to teamwork. The mediating role of the emergent state is supported by a recent study, where team interdependence was found to positively influence team behavioral integration, and the team emergent state was found to positively affect team performance (Zhang & Kwan, 2019). The same study also showed that team behavioural integration could mediate the relationship between team independence and team performance. However, these relationships have not been examined yet in the context of IPE, specifically with collective dedication as an outcome.

### Learning outcome: collective dedication

Dedication is an essential component of collaborative practice, particularly in IPE. In such collaborative settings, individual dedication translates into collective dedication when team members perceive that their team demonstrates a sense of significance, enthusiasm, inspiration, pride, and challenge (Salanova et al., 2003; Schaufeli et al., 2002). There is, however, a paucity of research on what influences collective dedication. From the perspective of SIT, collaboration is strengthened when there is positive interdependence among team members, such as when students believe that their contribution is essential to the team’s successful completion of the assigned activity. Moreover, self-determination theory argues that humans need to establish and maintain harmonious relationships with others, which is operationalized as relatedness (Deci & Ryan, 2000). Positive interdependence leads to a greater sense of relatedness, which increases the opportunities for collective dedication. Regarding the team emergent state, higher positive interdependence also leads to higher group potency and collective efficacy, which in turn leads to a higher collective dedication to teamwork in the IPE context.

### Interprofessional education and collaborative practice (IPECP)

The IPECP, the programme for which the current study was implemented (Supplementary Fig. 1), capitalizes on the merits of team-based (Michaelsen et al., 2002) and case-based learning (Thistlethwaite et al., 2012) pedagogies, which include the development of collaborative leadership, problem-solving, conflict resolution, and clinical reasoning skills. A three-part program spanning three weeks was designed using a COVID-19 Infection Control and Management simulation course. In Part 1 (Preparation, Online, One week), the interprofessional team develops cohesiveness and identity through various activities, including building rapport by meeting team members via a learning management system, reading the pre-class study materials (e.g., e-books, journal articles, websites, etc.) related to COVID-19, writing multiple-choice questions based on the pre-class study materials, and creating the team’s name. In Part 2 (Readiness Assurance Process and Application Exercise, In-person, Two weeks), students meet their teammates in person and take the same test twice, first individually and then as a team. The test was based on the pre-class study materials that the students read in Part (1) When taking the test as a team, they are presented with the opportunity to discuss each item and the individual members’ thought processes in determining the best answer. Through this process, the students learn how to engage in respectful communication and show recognition of others’ expertise. Then, in an application exercise, the interprofessional teams learn about a COVID-19 patient case and they are tasked to design an integrated interprofessional healthcare plan. Near-peer teachers (i.e., students who are at least one year more senior who teach more junior students; Bulte et al., 2007) facilitate discussion using the pedagogy of constructive controversy (Johnson et al., 2000, 2014). Finally, in Part 3 (Enrichment Activity, In-Person, One day), the teams gather in person to engage in dialogue with the content experts (i.e., an interprofessional team of experts on the COVID-19 case) about the answers to the test they took in Part (2) The teams will also receive feedback from the content experts about their interprofessional healthcare plans and share their reflections on their teamwork experience. Lastly, the announcement and presentation of winning teams as vetted by content experts for being the best interprofessional care management planning become the highlight of Part 3.

### The current study

Although previous research has examined the effects of some team characteristics (e.g., composition, size, and location) of interprofessional teams on patient health outcomes (Wranik et al., 2019), the impact of team interdependence, a team characteristic, has received little attention. Also, the mechanism through which positive interdependence and sense of relatedness, group potency, and collective efficacy may be related to one another in influencing collective dedication has not been specified and fully examined in past research.

Therefore, a more specific explanation of the mechanism between positive interdependence and collective dedication is needed to make IPE more effective. Taken together, this study aims to examine the relationships among team psychological factors (e.g., interdependence, relatedness, efficacy, and potency) and how they lead to collective dedication in a collaborative learning context. More specifically, it aims to investigate whether sense of relatedness (an individual’s motivational state), group potency, and collective efficacy (team emergent states) represent the potential mechanisms through which positive interdependence influences team collaboration performance. Our study variables were selected based on theoretical reasoning. Drawing from SIT, we identified positive interdependence as the input factor. Self-determination theory (Ryan & Deci, 2000) indicated sense of relatedness as a key motivational state. The IMO framework directed attention to collective efficacy and group potency as team emergent states, with collective dedication as the outcome. This theoretical integration guided our selection of validated instruments that appropriately measured these constructs.

We hypothesize that:

#### H1

Students’ positive interdependence predicts the sense of relatedness, collective efficacy, and group potency

#### H2

Students’ sense of relatedness predicts group potency, collective efficacy, and collective dedication

#### H3

Students’ group potency and collective efficacy predict collective dedication, and

#### H4

Collective efficacy mediates the relationship between group potency and collective dedication

An understanding of the confluence of individual motivation states and team emergent states is important in our effort to delineate the contribution of individual and team-level states to inform theory and practice in running IPE among health and social care students.

## Methods

This cross-sectional quantitative study was facilitated among health and social care undergraduate students enrolled in a credit-bearing interprofessional education (IPE) course. The ethics and procedures of this study were approved by the Human Research Ethics Committee for Non-clinical Faculties of the designated university (approval number EA210433) prior to the conduct of the survey.

### Study design and setting

This study was designed to investigate team psychological factors that could influence collaborative outcomes, such as collective dedication among health and social care students enrolled in a university in Hong Kong. Specifically, we measured these predictor and outcome variables using quantitative scales and ran statistical analyses using the data collected.

### Participants

The study involved a final sample of 236 health and social care undergraduate students (39.33% response rate) who volunteered to participate out of 600 students enrolled in a credit-bearing IPE. Using G*Power version 3.1 (Faul et al., 2009), we computed for a priori sample size with a given medium level of effect size at f2 = 0.15, power level at 0.95, and level of significance set at 0.05 on F tests of linear multiple regression with four predictors, the computed minimum sample size was 129. Among the final sample of students, 34% were males while 66% were females. They were from seven different programs (response rate per discipline in parentheses): Chinese medicine (*n* = 5 of 19, 26.31%), nursing (*n* = 86 of 201, 42.78%), pharmacy (*n* = 6 of 13, 46.15%), speech therapy (*n* = 16 of 36, 44.44%), social work (*n* = 10 of 32, 31.25%), law (*n* = 18 of 42, 42.85%), and medicine (*n* = 95 of 257, 36.96%). The average age was 21.3 years (*SD* = 1.40). Most of the participants were in their 5th year (76.69%).

### Conceptual framework

Built from our literature review of existing theoretical models, we conceptualized a model to elucidate the potential mechanisms that team psychological factors bring to the achievement of collective dedication in IPE (Fig. 1).

### Measures

#### Positive interdependence

We used a survey developed by Janssen et al. (1999) to measure students’ positive interdependence. This scale is composed of four items adapted to IPE (e.g., “*Characteristic for our IPE team was that goal attainment for one team member facilitated goal attainment for others*”) with a response format from 1 (strongly disagree) to 5 (strongly agree). Higher mean scores indicate greater positive interdependence. In the current study, the Cronbach’s alpha on this scale was 0.95.

#### Sense of relatedness

Sense of relatedness was measured using eight items from the subscale of the Basic Psychological Need Satisfaction in General (BPNS; Deci et al., 2001). We modified some items slightly to fit in the context of interprofessional team learning. For instance, item 2, “*I really like the people I interact with*,” was slightly modified to “*I really like my teammates I interact with in my healthcare team*.” Participants indicated their agreement with the questions, ranging from 1 (not at all true) to 7 (very true). Responses to the three negatively stated items (e.g., “*The teammates I interact with do NOT seem to like me much*”) were reverse-coded. Higher mean scores indicate a greater sense of relatedness. The internal reliability of the scale in the present study was 0.97.

#### Group potency

We used an eight-item survey developed by Guzzo et al. (1993) to evaluate students’ group potency (e.g., “*This team has confidence in itself*”). The scale has a five-point response format, from 1 (to no extent) to 5 (to a great extent). Higher mean scores indicate greater group potency. In the current study, the Cronbach’s alpha on this scale was 0.97.

#### Collective efficacy

Collective efficacy was measured using a four-item scale adapted version of the Generalized Self-Efficacy assessment (e.g., *“I feel confident about the capacity of the group to perform the tasks well*”; Salanova et al., 2003). Questions were designed on a five-point scale from 1 (very small extent) to 5 (very large extent). Higher mean scores indicate greater collective efficacy. The internal reliability of this scale in the present study is acceptable (α = 0.82).

#### Collective dedication

Collective dedication was evaluated using an adapted version of the Engagement Questionnaire (Salanova et al., 2003). This questionnaire is composed of four items (e.g., “*My group felt enthusiastic about the task*”) with a response scale of 1 (strongly disagree) to 5 (strongly agree). Higher mean scores indicate greater collective dedication. In the current study, the Cronbach’s alpha of this scale was 0.95.

### Data collection procedures

The ethics and procedures of this study complied in accordance with the ethical principles for conducting research involving human participants, consistent with the 1964 Helsinki Declaration and its later amendments. Ethics approval was given by the Human Research Ethics Committee for Non-clinical Faculties of the University of Hong Kong with approval number EA210433.

After the students’ participation in an IPE simulation course in October 2021, they were requested to complete an anonymous online survey in Qualtrics within a week. Following all the measures mentioned in the previous subsection, the students were presented with a total of 35 survey items. In the informed consent included in the online survey, students were informed that their participation in this study was completely voluntary, that their participation or non-participation would have no effect on their academic standing, and that an appropriate consent procedure was followed.

### Data analysis

​​All variables were included in the descriptive and inferential analyses. We acknowledge that Likert-scale data are technically ordinal. However, consistent with common practice in social science and health professions education research, we treated the data as interval-level for statistical analysis (Norman, 2010; Sullivan & Artino, 2013). Parametric statistics are robust with Likert scale data when scales have five or more response options and data approximate normal distributions (Norman, 2010). All our study variables demonstrated acceptable distributional properties (see Table 2).

**Table 2:** Descriptive statistics of model variables in the study using cross-sectional data from undergraduate health and social care students who participated in an interprofessional education simulation course (*n* = 236)

Variables	Positive interdependence	Collective efficacy	Relatedness	Group potency	Collective dedication
Maximum	5.00	5.00	7.00	5.00	5.00
Minimum	1.00	1.00	1.50	1.00	1.00
Mean	3.73	3.76	4.56	3.66	3.63
Cronbach’s alpha	0.95	0.82	0.97	0.97	0.95
*SD*	0.77	0.77	0.93	0.79	0.86
Skewness	-0.75	-0.90	0.34	-0.82	-0.78
Skewness (SE)	0.16	0.16	0.16	0.16	0.16
Kurtosis	1.36	1.73	0.74	1.58	0.96
Kurtosis (SE)	0.31	0.31	0.31	0.31	0.31

*Abbreviations: SD*, standard deviation; *SE*, standard error

The hypothesized relationships among the variables were tested using path analysis. It was considered an appropriate technique for testing the fit of a hypothesized model with the observed data through goodness-of-fit statistics (Byrne, 2001). The descriptive statistics, direct and indirect effects were analyzed using SPSS version 23, while the hypothesized model was analyzed through path analysis using maximum likelihood estimation in AMOS Version 26.0, which accounts for missing data. Confidence intervals (95%) were generated by bootstrapping with 5000 resamples. Model fit was assessed using the Comparative Fit Index (CFI), the Tucker-Lewis Index (TLI), the Root Mean Square Error of Approximation (RMSEA), and the Standardized Root Mean Square Residual (SRMR). A good model fit was indicated by a CFI of 0.90 or greater, a TLI of 0.90 or greater, and an SRMR of 0.08 or lower (Hu & Bentler, 1999).

## Results

### Descriptive statistics

The descriptive statistics are reported in Table 2. Participants’ mean scores on the study variables are the following: Positive interdependence (*M* = 3.37; *SD* = 0.77); Collective efficacy (*M* = 3.76; *SD* = 0.77); Relatedness (*M* = 4.56; *SD* = 0.93); Group potency (*M* = 3.66; *SD* = 0.79); and collective dedication (*M* = 3.63; *SD* = 0.86).

### Positive interdependence predicts sense of relatedness, collective efficacy, and group potency

Consistent with H_1,_ results from the path analysis revealed that students’ positive interdependence directly and positively predicts their sense of relatedness (β = 0.63, 95% CI [0.55, 0.71]), collective efficacy (β = 0.39, 95% CI [0.24, 0.54]), and group potency (β = 0.69, 95% CI [0.58, 0.80]).

### Sense of relatedness predicts group potency, collective efficacy, and collective dedication

Path analysis results further showed that students’ sense of relatedness directly and positively predicts their group potency (β = 0.24, 95% CI [0.13, 0.35]), collective efficacy (β = 0.09, 95% CI [0.01, 0.17]), and (β = 0.69, 95% CI [0.58, 0.80]). These results were consistent with our H_2_.

### Group potency and collective efficacy predict collective dedication

In addition to the abovementioned results, students’ group potency (β = 0.54, 95% CI [0.38, 0.71]) and collective efficacy (β = 0.24, 95% CI [0.08, 0.40]) both directly and positively predict collective dedication. These results support our H_3_.

### Collective efficacy mediates the relationship between group potency and collective dedication

Lastly, consistent with our H_4,_ our mediation results revealed that collective efficacy (β = 0.12, 95% CI [0.05, 0.22]) mediated the association between group potency and collective dedication. This positive indirect effect of collective efficacy means that higher group potency is linked with higher collective efficacy, which in turn, is linked with higher collective dedication.

**Fig. 2:**
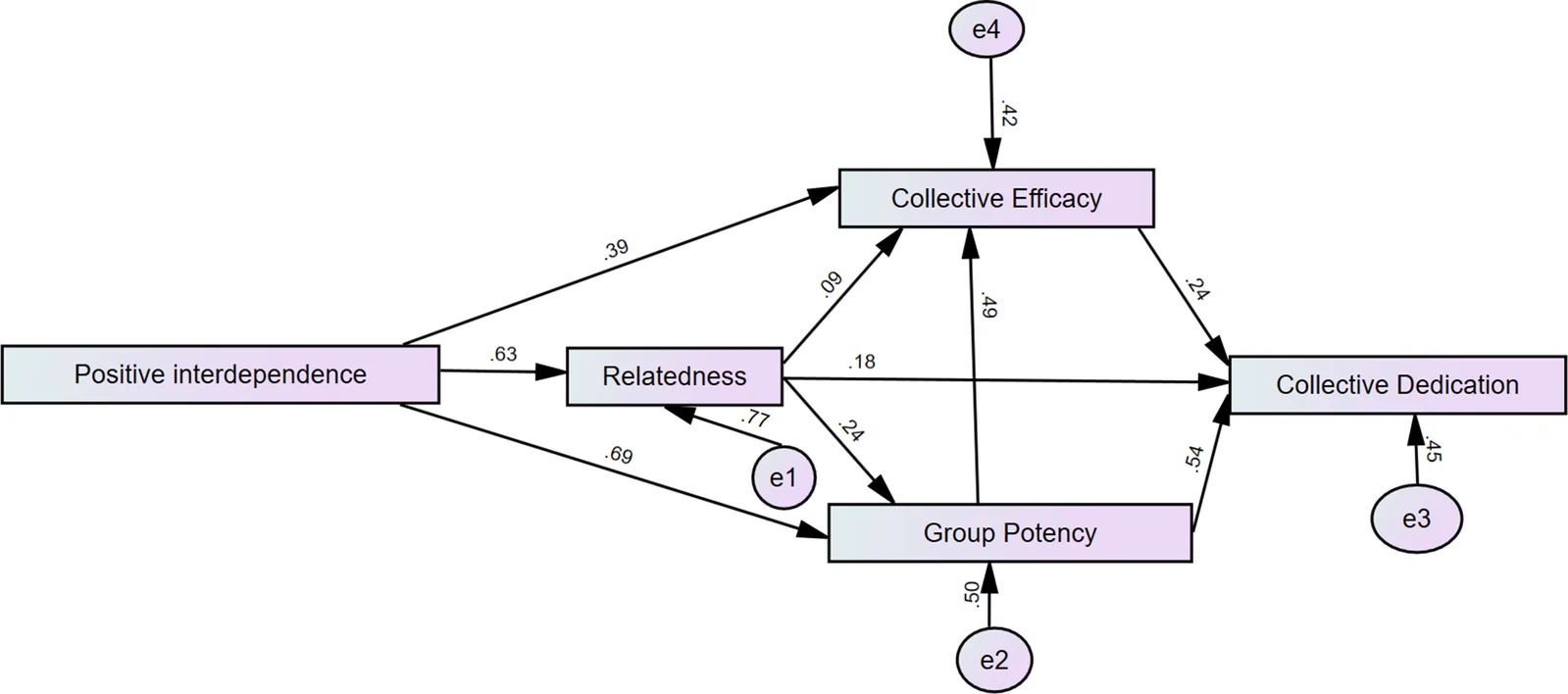
The derived model with significant path estimates at *p* < .05 using data from undergraduate health and social care students who participated in an interprofessional education simulation course (*n* = 236). All paths are positive

Overall, positive interdependence indirectly and positively predicts collective dedication via relatedness, group potency, and collective efficacy. The full hypothesized model (Fig. 2) that shows all the paths among the variables had an acceptable fit with the data, χ^2^ (1) = 2.85, *p* = .091, χ^2^/*df* = 2.85, CFI = 0.99, TLI = 0.99, RMSEA = 0.08, 90% CI [0.00, 0.22], SRMR = 0.01. Table 3 provides a summary of the direct, indirect, and total effects relationships among variables in the model.

**Table 3:** Standard direct, indirect, and total effects in the final model using cross-sectional data from undergraduate health and social care students who participated in an interprofessional education simulation course (*n* = 236)

Predictor	Criterion	Direct effect	Indirect effect	Total effect
Positive interdependence	Relatedness	0.63	0.00	0.63
	Group potency	0.69	0.15	0.85
	Collective efficacy	0.39	0.47	0.86
	Collective dedication	0.00	0.78	0.78
Sense of relatedness	Group potency	0.24	0.00	0.24
	Collective efficacy	0.09	0.12	0.21
	Collective dedication	0.18	0.18	0.36
Collective efficacy	Collective dedication	0.24	0.00	0.24
Group potency	Collective efficacy	0.49	0.00	0.49
	Collective dedication	0.54	0.12	0.66

## Discussion

The current study proposed a conceptual framework to describe the relationships among team psychological factors and examine the mechanisms that lead to the pathway to collective dedication in IPE. To the best of our knowledge, the relationships between positive interdependence, collective efficacy, sense of relatedness, group potency, and collective dedication among health profession students have not yet been fully clarified and modelled by previous studies in the context of interprofessional education. In response to this research gap, the current study developed a conceptual model based on the SIT and IMO frameworks to examine the mechanism for achieving collective dedication. Descriptive statistical results indicated that, in aggregate, the participants had an average to above-average range of mean scores across the study variables. On the other hand, the inferential statistical results indicated an indirect effect of positive interdependence on collective dedication through students’ sense of relatedness, group potency, and collective efficacy. All four hypotheses of the present study were supported by the findings.

### Positive interdependence and collective dedication: lens of SIT

We found that positive interdependence among health and social care students in IPE partially predicted their collective dedication via their sense of relatedness, group potency, and collective efficacy. In addition, positive interdependence was found to be positively linked with collective dedication and that sense of relatedness, group potency, and collective efficacy positively and partially mediate such a link. These findings provide evidence consistent with previous studies on the importance of psychological variables in collective dedication (e.g., Ganotice et al., 2021) In line with past studies (Ganotice et al., 2022; Shimizu et al., 2020), the findings also provide support for SIT as a useful theoretical framework to better understand how collaborative learning occurs in health care and social care education. Consistent with the principles of SIT, positive interdependence among group members promoted collaboration (Gully et al., 2002), as students believed that their contribution was critical to the group’s success in completing assigned tasks (Johnson & Johnson, 2009).

Positive interdependence leads to individual accountability, or a sense of responsibility for completing one’s work, as well as facilitating the work of other group members. It also leads to beneficial interaction, wherein students encourage and assist one another in achieving group goals (Johnson & Johnson, 2009). Our conceptual model supported the theoretical argument and extended the utility of the theory in the IPE realm. In particular, students who showed higher positive interdependence for goal attainment, identification of success, benefit, and gain, had a greater sense of relatedness in a team (individual motivation state), which in turn led to higher group potency and collective efficacy (team emergent states), and thus, perceived higher collective dedication (learning outcomes).

### The predictive role of sense of relatedness

We found that students’ sense of relatedness in the IPE context predicted collective efficacy, group potency, and collective dedication. This extends Ganotice et al. (2021)‘s finding on the positive relationship linking a sense of autonomy and collective dedication, wherein in the current study, a sense of relatedness predicted team effectiveness and goal achievement. The students’ sense of relatedness was found to increase their thriving at work in interprofessional learning by creating a caring environment within the group to foster a sense of belonging to sustain greater enthusiasm for team tasks (Ganotice et al., 2021). IPE is an ideal context for meeting students’ need for relatedness, as it allows students from various backgrounds to develop a sense of communion and close relationships. Students tend to perceive the team as more willing, enthusiastic, motivated, and involved in completing tasks when their sense of relatedness is fostered. The students who participated in the IPE program discussed pre-class materials and formulated health management plans. This strategy might have helped them find a sense of belonging in the team, which could greatly promote their involvement and enhance team dedication. The students’ sense of relatedness and collective dedication were mediated by group potency and collective efficacy. In team collaboration, students who trust others to “back them up” respond with more vigour and constructive actions and hence perceive higher collective efficacy and group potency. This encourages the students to be more committed and dedicated to the team.

### Collective efficacy: serving as the mediator between group potency and collective dedication

Collective efficacy served as a mediator between group potency and collective dedication. This is consistent with the finding of a meta-analysis demonstrating the positive relationship between collective efficacy and group potency (Stajkovic et al., 2009). Potent teams tend to be more positive and have a greater learning orientation (Costa et al., 2014; Van den Bossche et al., 2006). As a result, despite difficult circumstances and challenges, these teams typically persevere and work together as a team, and even strive to master new challenges (Stoverink et al., 2020). The current finding reaffirms that a team with higher potency tends to have more confidence in performing tasks. Furthermore, this is consistent with previous studies where collective efficacy has consistently been found to relate positively to team effectiveness (McLarnon & Woodley, 2021). IPE emphasizes the importance of establishing teamwork and building interprofessional collaboration (Thistlethwaite & Moran, 2010). Hence, the current finding supports this argument and focuses attention on another collaboration outcome besides team effectiveness: collective dedication, which is an underexplored outcome in the literature.

### The role of culture

Given that this study generally involved an Eastern sample, culture could potentially play a role in making sense of the findings. Previous investigations have highlighted that people from collectivist cultures (i.e., Eastern) tend to value the needs of the group or the society to which one belongs more than their own individual interests when compared with people in individualist cultures (i.e., Western; Markus & Kitayama, 1991, 1998). From our findings, it appears that our participants’ collectivist culture yields support to the assertion that students with high positive interdependence tend to have a high sense of relatedness, collective efficacy, and group potency that eventually leads to high collective dedication. Although studies on collective efficacy and group potency among interprofessional students in the Eastern context are still scarce, a previous comparison of the importance of relatedness among students in Eastern and Western cultures showed that students in both cultures put importance on relatedness (Nalipay et al., 2020). Taken altogether, belonging to a collectivist culture may play a role in the students’ positive interdependence, sense of relatedness, collective efficacy, group potency, and collective dedication. Culture’s further impact on such variables and samples is worthy of further investigation in the future.

### Study implications

The study’s findings have important implications. First, promoting team effectiveness by evoking the affective aspects is crucial but often overlooked. Recognizing and reinforcing the importance of dedication is the first step to designing an IPE program, which is also a promising direction for future research. Practically, fostering a shared sense of purpose and commitment among students from different professions is imperative. In this regard, start by creating a shared vision that emphasizes the importance of collaboration and teamwork in providing quality care to patients. This vision should be communicated to all students, and it should be reinforced throughout the IPE program.

Second, positive interdependence is integral to IPE. In collaborative learning activities, establishing shared goals and mutual accountability among team members can help promote positive interdependence. Anyone in the group who believes there is merit in working together and that individual learning and work products will be improved by collaboration is deemed to be engaging in positive interdependence, which is a prerequisite for collective dedication. An example of how educators could promote positive interprofessional interdependence is by designing group activities that are a combination of individual and team activities (e.g., self-taken tests and group-taken tests). Individual activities can build the self-efficacy of students on a given task and when they join a group, they may feel more confident in contributing their skills and ideas to their group in doing team activities. This may lead to more openness and trust within the group which could pave the way for each group member to realize that they have shared responsibility and success within the group. In addition, educators may also focus on designing assessment tools that can account for students’ contributions to team tasks and that such assessment criteria should be known and clear to each group member before doing the group tasks. This could aid in promoting positive interdependence as they would realize beforehand that their contributions would benefit not only themselves but the whole team.

Lastly, understanding the mechanism that promotes collective dedication among health professional IPE students can inform the design of an IPE program that fosters team effectiveness among future healthcare workers. The conceptual framework we proposed also illustrates that interprofessional learning could increase students’ motivation on an individual level by fostering a sense of relatedness and team motivation that results from collective efficacy and group potency. When team members have a strong sense of collective efficacy and group potency, they are more likely to be motivated to work together and achieve their shared goals, which in turn promotes collective dedication.

Extending these findings to a wider extent, developing these characteristics among interprofessional undergraduate students could potentially benefit not only their future patients but also their future colleagues in the healthcare workplace. For example, an interprofessional healthcare team with inherently high positive interdependence, collective efficacy, group potency, and collective dedication could result in more holistic patient care and fewer medical errors. Aside from medical errors, professional disagreements among healthcare professionals may also be mitigated when the same professionals have previous interprofessional training that immerses them in team activities that can promote respectful communication, teamwork, and psychological factors such as collective dedication.

### Study limitations and recommendations

To the best of our knowledge, this is the first study to simultaneously investigate the relationships between positive interdependence, the sense of relatedness, collective efficacy, group potency, and collective dedication in the context of IPE. Combining the two theoretical models (i.e., SIT and IMO), a new conceptual framework is proposed for exploring what and how the pertinent psychological factors can lead to collective dedication, which may shed light on future study direction and practical application in IPE. However, some limitations need to be acknowledged.

First, the study design is cross-sectional. As a result, causality cannot be established. Longitudinal or prospective studies are needed in the future to build a stronger directionality argument for examining the proposed model. Additionally, the three-week duration of our IPE course represents a relatively short period for team development. Our data collection captured team psychological factors at a specific point in the team development process, and the relationships we observed may vary across Tuckman’s (1965) stages of team development (forming, storming, norming, performing). Future longitudinal studies could examine how the pathways to collective dedication evolve across different developmental stages.

Second, data were gathered through students’ self-report scales. In the future, other types of measures could be considered (e.g., peer evaluation, teacher rating, qualitative measures) to have additional sources of data. Furthermore, future studies can consider enlisting students from multiple institutions to maximize the sample’s representativeness rather than enlisting students from a single university.

Third, given that this study was conducted in a largely collectivist culture, which generally prioritizes the needs and values of the group over individual interests (Markus & Kitayama, 1991, 1998), it may be possible that outcomes will be different in an individualist culture. Future studies can consider examining pathways to collective dedication among students from different cultures.

Fourth, while our model integrated SIT and IMO frameworks, we acknowledge that alternative theoretical perspectives could offer different insights into collective dedication. For instance, group development theory (Tuckman, 1965) might emphasize temporal dynamics that our cross-sectional design cannot capture, while transformational leadership theories could highlight facilitator roles we did not measure. We selected SIT and IMO for their specific relevance to collaborative learning contexts and their comprehensive framework from inputs to outcomes. Future research could examine competing theoretical models or incorporate additional perspectives to further the understanding of collective dedication in IPE.

Lastly, our IPE course included a competitive element wherein winning teams were recognized based on their interprofessional care management plans. While this competition was designed to enhance motivation and engagement, consistent with team-based learning pedagogy, it may have influenced team dynamics and the relationships among study variables. The interplay between collaboration and competition in educational settings is complex, and future studies could examine how different incentive structures (competitive vs. purely collaborative) affect the pathways to collective dedication in IPE.

## Conclusions

The current study extends our understanding of the pertinent pathways to collective dedication in the realm of IPE and health professions education. We found that positive interdependence had an indirect effect on collective efficacy via sense of relatedness, group potency, and collective efficacy. The study was able to contribute to the expansion of existing knowledge in medical education. Theoretically, building on the SIT and IMO, the proposed conceptual framework can be used for a deeper understanding of collaborative learning among healthcare teams. In practice, IPE implementers and course designers in health and social care education settings can consider emphasizing positive interdependence, individual relatedness, and the efficacy and potency of teamwork to enhance collective dedication in future IPE interventions. Promoting these factors during undergraduate years could instill collaborative capabilities and behaviors among the students which they can, in turn, bring to their respective healthcare fields that are now becoming in need of greater interprofessional collaboration as diseases are becoming increasingly complex to treat.

## Supplementary Information

Below is the link to the electronic supplementary material.

Supplementary Material 1

## Data Availability

The data that support the findings of this study can be requested from the corresponding author upon reasonable request.
