# Supplementary material for: What predicts collective dedication in health professions education? A path analysis among health and social care students
Source: Adv Health Sci Educ Theory Pract. 2025 Nov 3;31(4):1067–87. doi: 10.1007/s10459-025-10485-w (PMC13553669; doi:10.1007/s10459-025-10485-w)
Supplement: Supplementary file 1 [file 10459_2025_10485_MOESM1_ESM.docx]

**Supplementary Figure 1**

Independent

study

Readiness Assurance Process (tRAT)

Goal setting

-Post-

evaluation

-Peer/Self evaluation

Care planning

-Pre- evaluation survey,

-Team naming,

-Online discussion, MCQ writing

-Group reading of pre-class study materials

-Small group discussion

- Presentation of winners

-Whole class debriefing

- RAT Discussion

- Care Plan

Case-based learning

Reflection

PART 1

PART 2

PART 3

**Supplementary Figure 1.** The IPECP Model attended by the participants. Implemented at the University of Hong Kong during the academic year, 2021-2022.
